# Taurine alleviated paraquat-induced oxidative stress and gut-liver axis damage in weaned piglets by regulating the Nrf2/Keap1 and TLR4/NF-κB signaling pathways

**DOI:** 10.1186/s40104-025-01244-3

**Published:** 2025-08-18

**Authors:** Chen Chen, Min Qi, Weilong Zhang, Fanxing Chen, Zhihong Sun, Weizhong Sun, Wenjie Tang, Zhenguo Yang, Xuan Zhao, Zhiru Tang

**Affiliations:** 1 Laboratory for Bio-Feed and Molecular Nutrition, College of Animal Science and Technology, Southwest University, Chongqing, 400715 China; 2 Yunnan Animal Husbandry Station, Kunming, 650225 China; 3 Animal Breeding and Genetics Key Laboratory of Sichuan Province, Sichuan Animal Science Academy, Chengdu, 610066 China; 4 Livestock and Poultry Biological Products Key Laboratory of Sichuan Province, Sichuan Animtech Group Co., Ltd., Chengdu, 610066 China

**Keywords:** Antioxidant, Gut-liver axis, Immune response, Intestinal barrier, Lipopolysaccharide, Paraquat, Taurine, Weaned piglets

## Abstract

**Background:**

Oxidative stress can impair intestinal barrier function and cause liver damage, resulting in reduced animal productivity. Paraquat (PQ) induces significant oxidative stress in weaned piglets. The antioxidant, anti-inflammatory, and metabolic regulatory functions of taurine (Tau), a free amino acid that is widely distributed in the body, have been extensively studied. However, the mechanisms by which dietary Tau alleviates oxidative stress and gut-liver axis damage in weaned piglets remain unclear.

**Methods:**

Forty weaned piglets (20 males and 20 females; 6.41 ± 0.11 kg; 25 days old; Duroc × Landrace × Yorkshire) were used in a 2 × 2 factorial design to investigate the mechanism by which dietary Tau (0% or 0.4%) alleviates PQ-induced oxidative stress and gut-liver axis damage. We analyzed key biomarkers related to gut barrier function, mucosal damage repair, liver damage, gut-liver immunity, antioxidant capacity, systemic immune homeostasis, antioxidant levels, and gut microbiota diversity in piglets under normal and acute oxidative stress. In particular, we evaluated the coordinated regulation of gut-liver axis function mediated by Tau through the Nrf2/Keap1 (antioxidant) and TLR4/NF-κB (immune modulation) signaling pathways. Partial least squares path modeling and molecular docking were used to explore the intrinsic relationship between PQ, Tau, and the gut-liver axis.

**Results:**

PQ exposure impaired gut barrier function, increased the liver fibrosis area, and markedly affected gut microbial diversity (*P* < 0.05). Tau effectively alleviated PQ-induced oxidative stress by activating the Nrf2/Keap1 pathway and inhibiting the TLR4/NF-κB pathway. This enhanced gut barrier function, promoted mucosal repair, and significantly suppressed the concentration and circulation of lipopolysaccharides in the blood, consequently reducing liver damage (*P* < 0.05). This further facilitated the optimization of gut microbiota composition, thereby supporting the positive regulation of the gut-liver axis and improving systemic immune and antioxidant functions.

**Conclusions:**

Tau improved the health status of weaned piglets under both normal and stressed conditions by modulating the Nrf2/Keap1 and TLR4/NF-κB pathways, offering a potential new nutritional strategy for alleviating gut-liver damage.

**Supplementary Information:**

The online version contains supplementary material available at 10.1186/s40104-025-01244-3

## Background

Modern intensive farming practices increase the risk of oxidative stress in weaned piglets [1]. Oxidative stress often leads to intestinal barrier dysfunction and liver impairment, which reduce production efficiency and impose an additional economic burden on the swine industry [2, 3]. The application of antioxidant feed additives is considered a nutritional strategy to promote the gut-liver axis health and growth in piglets.

The intestine and liver are the primary organs of the gut-liver axis. They collectively maintain animal health and production efficiency through bidirectional crosstalk within the gut-liver axis [4]. The gut microbiota, an integral part of both the intestinal barrier and the gut-liver axis, affects the intestine and liver through various mechanisms [5]. Impaired intestinal barrier function leads to microbial translocation. This allows exogenous toxins, lipopolysaccharides (LPS), and harmful bacteria to enter the liver via the portal vein, resulting in liver damage [6]. Similarly, liver damage disrupts bile acid secretion, thereby affecting the balance of the gut microbiota and leading to intestinal barrier dysfunction [7]. An imbalance in the gut-liver axis leads to intestine and liver damage, resulting in metabolic dysregulation and negatively affecting animal production efficiency.

Paraquat (PQ) is a fast-acting, nonselective herbicide that causes varying degrees of poisoning through contact, inhalation, or ingestion. PQ (8 mg/kg body weight, BW) is typically used to induce oxidative stress and inflammatory injury in piglets [8, 9]. It triggers oxidative stress by generating free radicals and reactive oxygen species (ROS), while simultaneously activating host immune responses and promoting the secretion of pro-inflammatory cytokines. The liver is a primary target of PQ toxicity, which is characterized by intestinal barrier disruption, inflammatory infiltration, liver fibrosis, and even organ failure [10, 11]. Oxidative imbalance primarily activates two sensitive signaling pathways: Nrf2/Keap1 and TLR4/NF-κB [12]. Activation of the Nrf2 pathway enhances antioxidant enzyme activity, whereas that of the NF-κB pathway promotes proinflammatory cytokine expression. Therefore, Nrf2/Keap1 and NF-κB are key targets for oxidative tissue injury [13]. PQ exposure activated the NF-κB signaling pathway, significantly increasing the secretion of pro-inflammatory cytokines, such as TNF-α, IL-1β, and IL-6, thereby leading to cellular and tissue damage [14, 15]. Although the specific pathways affected by PQ remain unclear, the PQ-induced model of oxidative stress and gut-liver axis injury provides a reliable experimental basis for evaluating nutritional interventions and underlying mechanisms.

Taurine (Tau), a semi-essential amino acid in mammals [16], possesses powerful antioxidant, anti-inflammatory, and immunomodulatory properties [17]. This amino acid binds to TLR4 and inhibits the TLR4/NF-κB pathway, ultimately reducing the levels of pro-inflammatory cytokines and suppressing intestinal inflammation [18]. In our previous study, supplementing piglet diets with 0.4% Tau alleviated intestinal oxidative damage, significantly improving growth performance and antioxidant capacity. Furthermore, it showed the potential to support intestinal barrier integrity and influence gut microbiota composition [19]. Therefore, Tau is an ideal candidate for protecting weaned piglets from oxidative stress. However, the mechanism through which Tau alleviates gut-liver axis damage and promotes health via the modulation of Nrf2/Keap1 and TLR4/NF-κB signaling pathways remains unclear and requires further investigation.

Based on these insights, we hypothesized that Tau alleviates PQ-induced oxidative stress and gut-liver axis damage by regulating Nrf2/Keap1 and TLR4/NF-κB signaling. Thus, our analyses provide a theoretical basis for optimizing antioxidant nutritional strategies in swine production.

## Materials and methods

### Animals, experimental design

Forty 25-d weaned piglets (Duroc × Landrace × Yorkshire; equal numbers of males and females) were obtained from Chongqing Nongdianshan Agricultural Technology Development Co., Ltd. (Chongqing, China). The animals underwent a 3-day adaptation period before experiments were conducted using a 2 (with or without Tau) × 2 (saline or PQ injection) factorial design. All piglets were weighed (6.41 ± 0.11 kg) and randomly assigned to four treatment groups (*n* = 10/treatment) based on initial body weight: control (NS), Tau-added (TNS), PQ-challenged control (NSP), and PQ-challenged and Tau-added (TNSP). Tau (purity > 99%) was purchased from Shanghai Yuanju Biotechnology Co., Ltd. (Shanghai, China), and PQ (methyl viologen, 98%) from Chengdu Huaxia Chemical Reagent Co., Ltd. (Chengdu, China). A 12-mg/mL PQ stock solution was prepared in saline (Shanghai Yuanye Bio-Technology Co., Ltd., Shanghai, China). The piglets were fed two diets with Tau levels of 0% and 0.4% for the first 28 d of the experiment (two treatment groups were fed each diet). Eight piglets with similar body weight were selected from each group on d 29 (*n* = 8/treatment). The two PQ-challenged groups received a single intraperitoneal injection of PQ (10 mg/kg BW), whereas the other two groups were injected with an equivalent volume of saline. After 3 d, blood was collected from the anterior vena cava of five piglets randomly selected from the eight piglets in each group before 07:00 on d 32 (*n* = 5 piglets/treatment). At 08:00, all 20 blood-sampled piglets (*n* = 5/treatment group) were euthanized for tissue collection. The formal experiment lasted 31 d. Piglets were weighed before the morning feed on d 29 and d 32. The daily feed intake was recorded throughout the study period. Furthermore, the average daily gain (ADG), average daily feed intake (ADFI), and feed conversion ratio (FCR) of piglets were calculated at different experimental stages.

Throughout the experiment, piglets were housed individually in pens measuring 1.5 m × 0.5 m × 0.8 m, with the ambient temperature maintained at 28 °C. Food and water were provided ad libitum. The basal diet (Additional file 1: Table S1a) was formulated based on a study by Li et al. [20] and practical production experience. All experimental procedures and animal care protocols were supervised and approved by the Institutional Animal Care and Use Committee (IACUC) of Southwest University (approval number: IACUC-20231001-02). Our study design, analysis, and reporting were conducted in accordance with the 3R principle (Replacement, Reduction, and Refinement) to minimize animal suffering and reduce the number of animals used.

### Sample collection

On d 32, 10 mL of blood was collected from each of the five piglets per group before the morning feed. The blood samples were allowed to clot at room temperature, followed by serum separation through centrifugation at 3,500 × *g* for 15 min at 4 °C using an Eppendorf 5418R centrifuge (Eppendorf AG, Hamburg, Germany). Samples were subsequently stored at −80 °C for biochemical and ELISA analyses. Following blood collection, the piglets were anesthetized via an intravenous injection of propofol at 3 mg/kg BW (Guangdong Jiabo Pharmaceutical Co., Ltd., Guangdong, China) and euthanized by exsanguination. Segments (3 cm each) of the jejunum and ileum, as well as 2 cm × 2 cm liver samples, were collected and fixed in 4% paraformaldehyde solution (Servicebio, Beijing, China) for tissue section preparation and morphological observation. After flushing the ileum with cold saline (Shanghai Yuanye Bio-Technology Co., Ltd., Shanghai, China), the mucosa was gently scraped using a glass slide, snap-frozen in liquid nitrogen, and stored at −80 °C for RT-qPCR and western blot analyses. Liver tissues were also snap-frozen in liquid nitrogen and stored at −80 °C for ELISA and RT-qPCR analyses. Moreover, ileal contents were snap-frozen in liquid nitrogen and stored at −80 °C for 16S rDNA sequencing analysis.

### Biochemical analysis and ELISA

The concentrations or activities of catalase (CAT), glutathione peroxidase (GSH-Px), total antioxidant capacity (T-AOC), hydroxyl radical scavenging activity (OH•^−^), superoxide anion scavenging activity (O_2_•^−^), superoxide dismutase (SOD), malondialdehyde (MDA), nitric oxide (NO), alkaline phosphatase (AKP), aspartate aminotransferase (AST), alanine aminotransferase (ALT), lactate dehydrogenase (LDH), gamma-glutamyl transferase (γ-GT), and total bilirubin (TBIL) were assayed according to the manufacturers’ instructions of the relevant biochemical kit (Nanjing Jiancheng Bioengineering Institute, Nanjing, China).

The concentrations of immunoglobulins (IgA, IgM, and IgG), cytokines (IL-6, IL-1β, TNF-α, IL-10, and IL-12), transforming growth factor-β1 (TGF-β1), diamine oxidase (DAO), D-lactic acid (D-LA), endotoxin (LPS), and prostaglandin E2 (PGE2) were determined using ELISA kits (Quanzhou Ruixin Biotechnology Co., Ltd., Quanzhou, China).

### H&E and Masson staining

Jejunum, ileum, and liver tissues were embedded in paraffin, deparaffinized, stained with hematoxylin and eosin (H&E), and sealed with neutral gum. Tissue morphology was observed using the TissueFAXS Plus system (TissueGnostics GmbH, Vienna, Austria). The intestinal villus height (VH) and crypt depth (CD) were measured using TissueFAXS Viewer (TissueGnostics GmbH, Vienna, Austria) with a double-blind analysis, with each slide observed five times. Lymphocytes and goblet cells were counted in five randomly selected fields per section for each piglet using ImageJ (National Institutes of Health, Bethesda, MD, USA).

Liver sections were stained with Masson's trichrome staining kit (Solarbio Life Sciences, Beijing, China), and images were captured using the TissueFAXS Plus system (TissueGnostics GmbH, Vienna, Austria). For quantification, images of the entire tissue area were collected, and the liver fibrosis area was analyzed using the ImageJ software (National Institutes of Health).

### Real-time qPCR

The primer sequences for the genes in the ileal mucosa and liver are listed in Table S1b. Total RNA extraction, reverse transcription, and real-time PCR were performed as previously described [21]. Glyceraldehyde-3-phosphate dehydrogenase was used as a reference gene. The expression levels of target genes related to intestinal barrier integrity (*OCLN*, *CLDN1*, *TJP1*, *MUC2*, and *MUC4*), mucosal repair (*VIL1*, *LGR5*, *BMI1*, *PCNA*, *CTNNB1*, *TCF4*, and *CCND1*), antioxidant activity (*NFE2L2*, *KEAP1*, *HMOX1*, *NQO1*, *GCLC*, *SOD2*, and *GPX4*), and immune function (*TLR4*, myeloid differentiation primary response 88 (*MyD88)*, *RELA*, *TNF*, *IL1B*, *IL6*, *TGFB1*, and *IL10*) were quantified using the 2^−ΔΔCt^ method, with relative fold changes calculated against the NS group.

### Western blotting assay

Total protein was extracted from ileal mucosa samples (0.03 g) using 300 μL RIPA lysis buffer (Shanghai Weiao Biotechnology Co., Ltd., Shanghai, China) and separated via sodium dodecyl-sulfate-polyacrylamide gel electrophoresis. The proteins were transferred onto a polyvinylidene fluoride (PVDF) membrane (Merck Millipore, Burlington, MA, USA), incubated overnight at 4 °C with primary antibodies, and then incubated at 25 °C for 60 min with secondary antibody. The following primary antibodies were obtained from Wuhan Sanying Biotechnology Co., Ltd. (Wuhan, Hubei, China): β-actin (1:10,000, 81115-1-RR), Nrf2 (1:3,000, 80593-1-RR), Keap1 (1:5,000, 10503-2-AP), Occludin (1:10,000, 27260-1-AP), ZO-1 (1:10,000, 21773-1-AP), β-catenin (1:10,000, 51067-2-AP), TCF4 (1:10,000, 22337-1-AP), IL-6 (1:3,000, 66146-1-Ig), NF-κB p65 (1:10,000, 80979-1-RR), and TGF-β1 (1:2,000, 21,898-1-AP). The secondary antibody used was a horseradish peroxidase-conjugated goat anti-rabbit antibody (1:6,000, RGAR001). The PVDF membrane was incubated with Tanon™ Femto-sensitive enhanced chemiluminescence reagent (Tanon, Shanghai, China), and the signal was captured using the ChemiDoc™ XRS + chemiluminescence imaging system (Bio-Rad Laboratories, Inc., Hercules, CA, USA). Band intensities were quantified using ImageJ (National Institutes of Health).

### Analysis of 16S rDNA gene sequencing

Total DNA was extracted from the samples using the Magbead Fecal Genomic DNA Extraction Kit (BioTeke Biotechnology Co., Ltd., Beijing, China) and quantified using a Qubit (Invitrogen, Waltham, MA, USA). PCR amplification was performed using universal primers 341 F (5′-CCTACGGGNGGCWGCAG-3′) and 805R (5′-GACTACHVGGGTATCTAATCC-3′) with the following cycling conditions: initial denaturation (98 °C, 30 s), 32 cycles (98 °C, 10 s; 54 °C, 30 s; 72 °C, 45 s), and final extension (72 °C, 10 min). The PCR products were purified using AMPure XP beads (Beckman Coulter Genomics, Danvers, MA, USA) and quantified using Qubit (Invitrogen). Purified PCR products were assessed using an Agilent 2100 Bioanalyzer (Agilent Technologies, Santa Clara, CA, USA) and an Illumina Library Quantification Kit (Kapa Biosciences, Woburn, MA, USA). The qualified sequencing libraries were diluted and pooled based on the required sequencing depths. The pooled libraries were denatured into single strands using NaOH and sequenced on an Illumina NovaSeq 6000 platform (LC-Bio Technology Co., Ltd., Hangzhou, China) with 2 × 250 bp paired-end reads.

### Molecular docking

The molecular structure of Tau (CID number: 72307) was obtained from PubChem (https://pubchem.ncbi.nlm.nih.gov/) [22]. The 3D coordinates for LRP6 (Protein Data Bank [PDB] ID: 3S8Z, resolution: 2.60 Å), Keap1 (PDB ID: 4IFJ, resolution: 1.80 Å), and TLR4 (PDB ID: 3VQ2, resolution: 2.48 Å) were downloaded from PDB (http://www.rcsb.org/). The protein and ligand files were first converted to the PDBQT format, all water molecules were removed, and polar hydrogen atoms were added. The grid box was centered to cover the domain of each protein and allow for free molecular movement. The binding pocket was set as a square pocket (30 Å × 30 Å × 30 Å) with a grid spacing of 0.05 nm. Molecular docking was conducted using Autodock Vina 1.2.2 (http://autodock.scripps.edu/) for model visualization.

### Data processing and statistical analysis

All statistical analyses and data visualizations were performed using R v4.3.3 (R Foundation for Statistical Computing, Vienna, Austria). Data are expressed as the mean and standard error of the mean (SEM). The main and interaction effects were assessed using two-way analysis of variance, and Tukey’s test was applied for significant interactions. Spearman’s correlation was used for continuous data, whereas point-biserial correlations were used for binary data. Correlation was classified as weak (|*r*| < 0.33), moderate (0.33 ≤ |*r*| < 0.66), or strong (|*r*| ≥ 0.66) (LinkET package). Partial least squares path modeling (PLS-PM) was conducted, with goodness-of-fit values classified as poor (0.2–0.5), acceptable (0.5–0.7), and excellent (≥ 0.7) using the plspm package. Data were visualized using the ggplot2 package.

## Results

### Effects of dietary Tau levels and PQ challenge on the growth performance of piglets

During the first 28 d, addition of 0.4% dietary Tau increased the ADG by 9.5% and reduced FCR by 9.3% compared to 0% dietary Tau treatment (*P* < 0.05, Table 1). Over the 3 d following PQ challenge, PQ exposure decreased ADG and increased FCR (*P* ≤ 0.017), whereas Tau addition increased ADG and reduced FCR compared to the diet without Tau (*P* ≤ 0.001). No significant interaction was observed between dietary Tau and PQ exposure on growth performance (*P* > 0.05).

**Table 1:** Effects of dietary Tau levels and PQ challenge on growth performance in weaned piglets

**Item**	− **PQ**		** + PQ**		**SEM**	***P***		
	**Tau 0%**	**Tau 0.4%**	**Tau 0%**	**Tau 0.4%**		**Tau**	**PQ**	**Tau × PQ**
1–28 d (*n* = 20)								
Initial weight, kg	6.51	6.33			0.263	0.605		
Final weight, kg	12.34	12.73			0.300	0.786		
ADG, kg	0.21	0.23			0.005	0.042		
ADFI, kg	0.51	0.51			0.016	0.780		
FCR, kg/kg	2.46	2.23			0.084	0.033		
28–31 d (*n* = 8)								
Initial weight, kg	12.36	12.53	12.33	12.48	0.141	0.903	0.577	0.970
Final weight, kg	13.12	13.36	12.99	13.21	0.148	0.659	0.462	0.966
ADG, kg	0.25	0.28	0.22	0.24	0.004	< 0.001	< 0.001	0.908
ADFI, kg	0.53	0.55	0.56	0.53	0.008	0.738	0.902	0.128
FCR, kg/kg	2.10	2.03	2.54	2.21	0.052	0.001	0.017	0.124

− PQ, sterile physiological saline (0.9% NaCl) was delivered intraperitoneally; + PQ, intraperitoneal injection of paraquat at a dose of 10 mg/kg body weight; Tau 0%, dietary taurine level 0%; Tau 0.4%, dietary taurine level 0.4%; *ADG* Average daily gain, *ADFI* Average daily feed intake, *FCR* Feed conversion ratio, *SEM* Pooled standard error of the mean

### Effects of dietary Tau levels and PQ challenge on intestinal mucosal barrier integrity and function in piglets

**Fig. 1:**
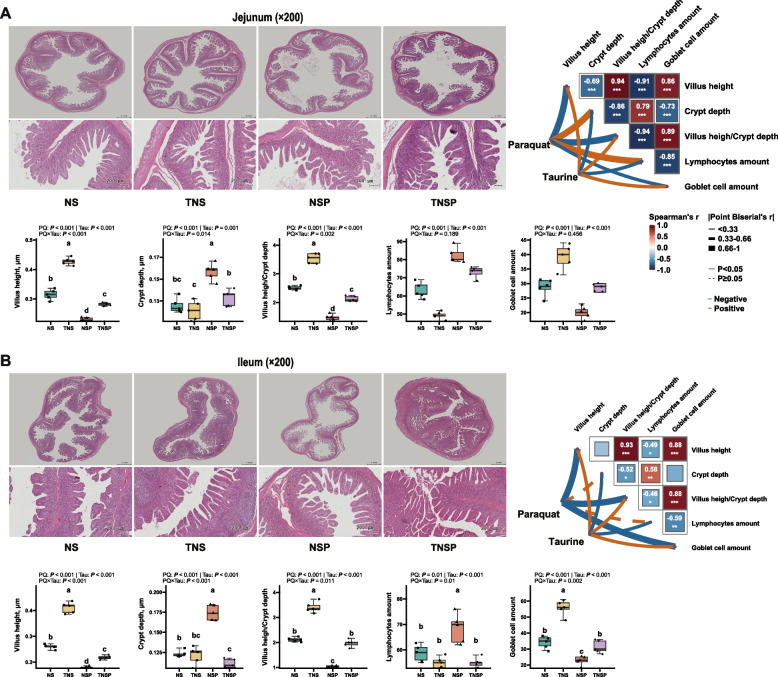
Intestinal morphology (*n* = 5). The panoramic and local tissue morphology of the jejunum (**A**) and ileum (**B**) (HE staining, × 200) and their correlation with Tau or PQ. Solid lines indicated *P* < 0.05, dashed lines indicated *P* > 0.05; lines from thin to thick represented correlation coefficients of |*r*| < 0.33, 0.33 ≤ |*r*| < 0.66, and 0.66 ≤ |*r*| < 1. Blue lines showed negative correlations, and orange lines showed positive correlations (The same applied below). NS: Piglets fed the basal diet (control group). TNS: Piglets fed the basal diet with the addition of 0.4% (w/w) Tau. NSP: Piglets fed the basal diet and challenged with PQ. TNSP: Piglets fed the basal diet with the addition of 0.4% (w/w) Tau and challenged with PQ (similarly hereafter). Means without a common superscript are significantly different (*P* < 0.05)

PQ exposure led to significant villus breakage and shedding and reduced villus density, whereas Tau alleviated these effects (Fig. 1A and B). Compared to non-PQ-exposed piglets, PQ exposure reduced the VH, VH/CD, and goblet cell counts. Tau increased VH, VH/CD, and goblet cell numbers compared to the diet without Tau (*P* < 0.001). A significant PQ × Tau interaction affected VH, CD, and VH/CD, with Tau improving VH and VH/CD and reducing CD in PQ-exposed piglets (*P* < 0.05).

**Fig. 2:**
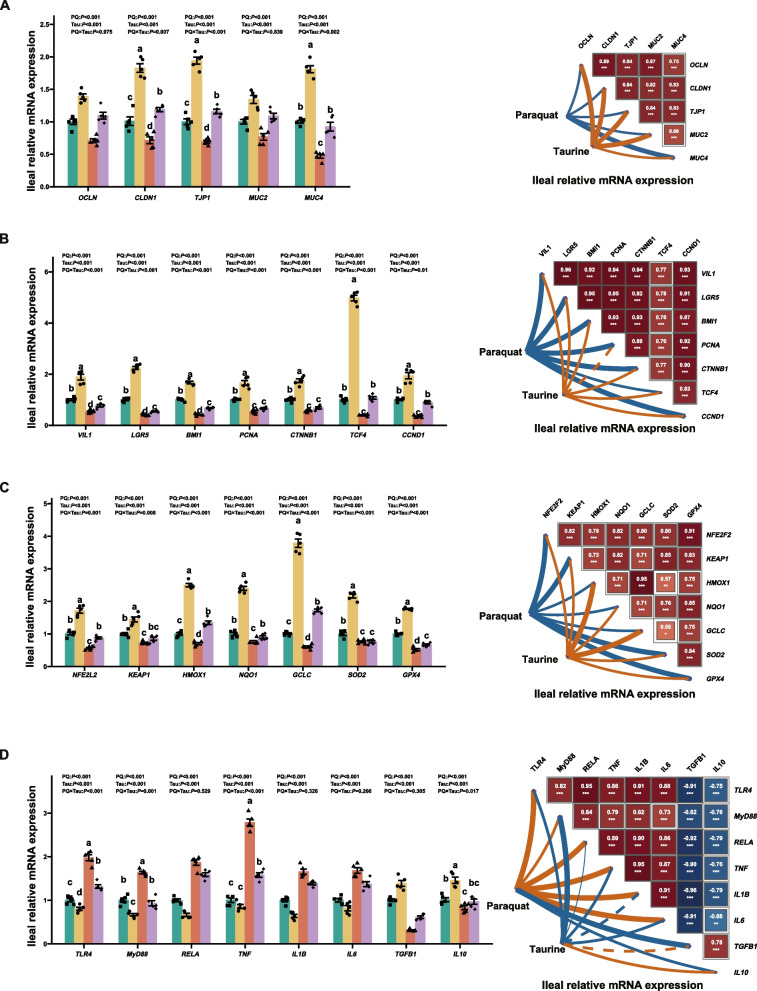
Gene expression in the ileal mucosa of weaned piglets (*n* = 5). **A** The mRNA expression of tight junction proteins and mucin, and their correlations with Tau or PQ. **B** The mRNA expression related to intestinal cell renewal, differentiation, and proliferation, and their correlations with Tau or PQ. **C** The mRNA expression involved in the antioxidant pathway and their correlations with Tau or PQ. **D** The mRNA expression involved in the immune-inflammatory pathway and their correlations with Tau or PQ. Means without a common superscript are significantly different (*P* < 0.05)

PQ exposure downregulates *OCLN*, *CLDN1*, *TJP1*, *MUC2*, and *MUC4* mRNA expression in the ileum. In contrast, Tau upregulated *OCLN*, *CLDN1*, *TJP1*, *MUC2*, and *MUC4* mRNA expression (*P* < 0.001, Fig. 2A). A significant PQ × Tau interaction was observed for *CLDN1*, *TJP1*, and *MUC4* (*P* ≤ 0.002), where Tau effectively reversed the PQ-induced reductions in these markers. Furthermore, PQ suppressed Wnt/β-catenin pathway genes and those for cell renewal, proliferation, and differentiation (*P* ≤ 0.001, Fig. 2B). Tau upregulated the expression of these genes compared to the diet without Tau (*P* < 0.001). A significant PQ × Tau interaction affected *VIL1*, *LGR5*, *BMI1*, *PCNA*, *CTNNB1*, and *TCF4* expression (*P* < 0.001), whereas Tau reversed the PQ-induced reduction in *VIL1*, *LGR5*, *BMI1*, and *TCF4* expression.

PQ reduced the mRNA expression of Nrf2-related antioxidant genes in the ileum (*P* < 0.001; Fig. 2C). However, Tau addition increased their expression compared to diets without Tau (*P* < 0.001). A significant interaction between PQ and Tau was observed for *NFE2L2*, *KEAP1*, *HMOX1*, *NQO1*, *GCLC*, *SOD2*, and *GPX4* (*P* < 0.012). Tau addition significantly reversed the PQ-induced reduction in *NFE2L2*, *HMOX1*, *GCLC*, and *GPX4* expression in PQ-challenged piglets. Moreover, PQ downregulated the expression of TLR4/NF-κB pathway genes and pro-inflammatory cytokines while upregulating that of anti-inflammatory cytokines (*P* < 0.001, Fig. 2D). Compared to diets without Tau, the addition of Tau reduced the inflammatory pathway and pro-inflammatory cytokine expression, while increasing the expression of anti-inflammatory cytokines (*P* < 0.001). A significant interaction between PQ and Tau was observed for *TLR4*, *MyD88*, *TNF*, and *IL10* (*P* ≤ 0.05). Tau significantly reversed the PQ-induced increase in *TLR4*, *MyD88*, and *TNF* in PQ-challenged piglets.

**Fig. 3:**
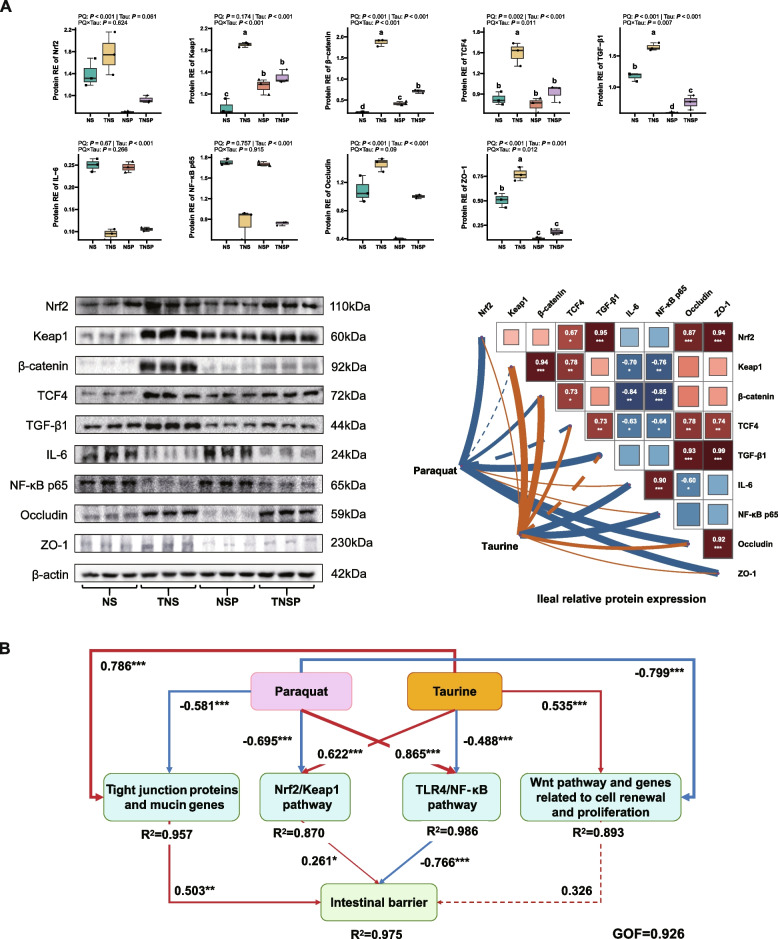
Protein expression in the ileal mucosa and PLS-PM analysis of gut barrier regulation (*n* = 3). **A** The expression of key proteins in the ileal mucosa and their correlations with Tau or PQ in piglets. Means without a common superscript are significantly different (*P* < 0.05). **B** The PLS-PM revealed the regulatory mechanisms of dietary Tau and PQ challenge on the gut barrier function in piglets. Significant paths (*P* < 0.05) were represented by solid lines, while dashed lines indicated non-significant paths (*P* ≥ 0.05). Orange and blue lines denoted positive and negative effects, respectively. The width of the lines reflected the path coefficients, and *, **, and *** indicated *P*-values of less than 0.05, 0.01, and 0.001, respectively (The same applies hereafter)

At the protein level, PQ reduced the expression of Nrf2, TCF4, TGF-β1, Occludin, and ZO-1 in the ileum (*P* ≤ 0.002). Compared to diets without Tau, Tau addition increased the expression of Keap1, β-catenin, TCF4, TGF-β1, Occludin, and ZO-1 while decreasing that of IL-6 and NF-κB (*P* < 0.001, Fig. 3A). A significant interaction between PQ and Tau was observed for Keap1, β-catenin, TCF4, TGF-β1, and ZO-1 (*P* ≤ 0.012). Tau addition significantly reversed the PQ-induced reduction in β-catenin and TGF-β1 expression in PQ-challenged piglets.

PLS-PM analysis was used to explore the relationships between PQ, Tau, and intestinal barrier function (Fig. 3B). PQ negatively affected the expression of tight junction proteins and mucin genes (β = −0.581), the Nrf2/Keap1 pathway (β = −0.695), the Wnt/β-catenin pathway, and genes related to intestinal cell renewal (β = −0.799), while positively affecting the TLR4/NF-κB pathway (β = 0.865). Tau exerted opposite effects (*P* < 0.001). Specifically, the Nrf2/Keap1 pathway (β = 0.261) and the expression of tight junction proteins and mucin genes (β = 0.503) positively affected the intestinal barrier, whereas the TLR4/NF-κB pathway (β = −0.766) had a negative effect (*P* < 0.05).

### Effects of dietary Tau levels and PQ challenge on serum biomarkers reflecting gut-liver health and systemic effects in piglets

**Fig. 4:**
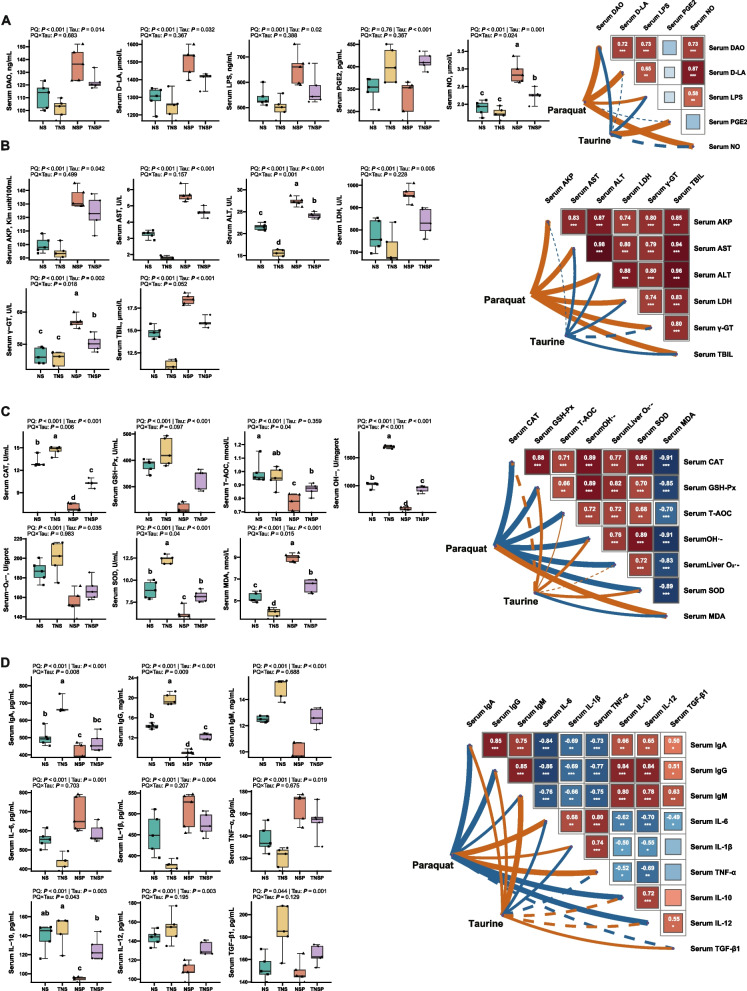
Serum biomarkers in piglets (*n* = 5). Biomarkers indicating gut barrier permeability and damage (**A**), liver damage (**B**), as well as the antioxidant (**C**) and immune (**D**) capacities of the body, and their correlations with Tau or PQ. Means without a common superscript are significantly different (*P* < 0.05)

PQ elevated the serum LPS, DAO, D-LA, and NO levels (*P* < 0.001; Fig. 4A). Tau reduced these factors and increased PGE2 levels compared to the diet without Tau (*P* < 0.05). A significant interaction between PQ and Tau was observed for NO, with Tau reducing NO levels in PQ-challenged piglets (*P* < 0.05). In addition, PQ increased serum AKP, AST, ALT, LDH, γ-GT, and TBIL levels (*P* < 0.001, Fig. 4B). Tau lowered these markers compared to the diet without Tau (*P* < 0.05). A significant interaction between PQ and Tau was observed for ALT and γ-GT, with Tau reducing their levels in PQ-challenged piglets (*P* < 0.05).

PQ decreased serum CAT, GSH-Px, T-AOC, OH•^−^, O_2_•^−^, and SOD levels, while increasing MDA levels (*P* < 0.001, Fig. 4C). Tau increased the levels of these antioxidants and reduced PGE2 levels compared to the diet without Tau (*P* < 0.05). Moreover, a significant interaction between PQ and Tau was observed for CAT, GSH-Px, T-AOC, OH•^−^, O_2_•^−^, MDA, and SOD. Tau increased serum CAT, T-AOC, OH•^−^, and SOD levels, while reducing MDA levels in PQ-challenged piglets (*P* < 0.05). PQ decreased serum IgA, IgG, IgM, IL-10, IL-12, and TGF-β1 levels, while increasing IL-6, IL-1β, and TNF-α expression (*P* < 0.05, Fig. 4D). In contrast, supplementation with Tau increased the levels of anti-inflammatory cytokines and decreased those of pro-inflammatory cytokines compared to the diet without Tau (*P* < 0.05). A significant interaction between PQ and Tau was observed in serum IgA, IgG, and IL-10 levels. In particular, Tau increased IgG and IL-10 levels in PQ-challenged piglets (*P* < 0.05).

### Effects of dietary Tau levels and PQ challenge on liver Injury and function in piglets

**Fig. 5:**
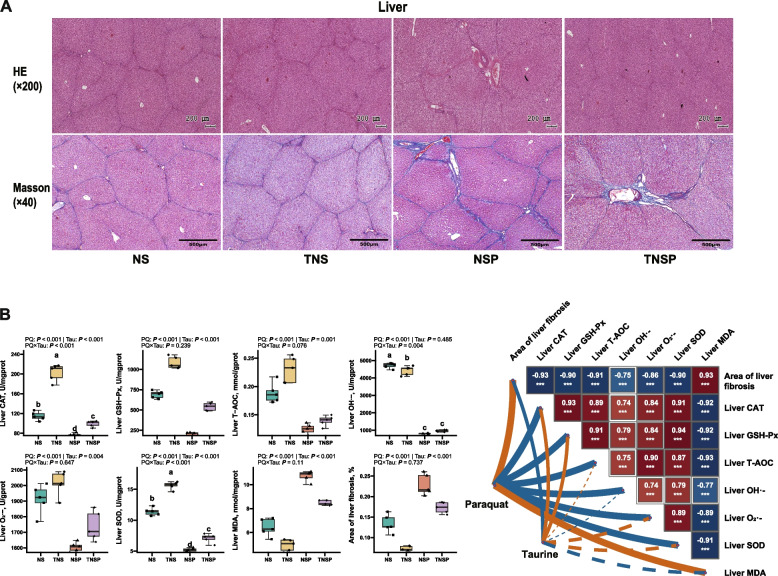
Liver morphology and antioxidant parameters in piglets (*n* = 5). **A** The liver tissue sections (HE, × 200; Masson, × 40) and their correlations with Tau or PQ. **B** The liver fibrosis area in piglets, the content of antioxidant factors in the liver, and their correlations with Tau or PQ. Means without a common superscript are significantly different (*P* < 0.05)

**Fig. 6:**
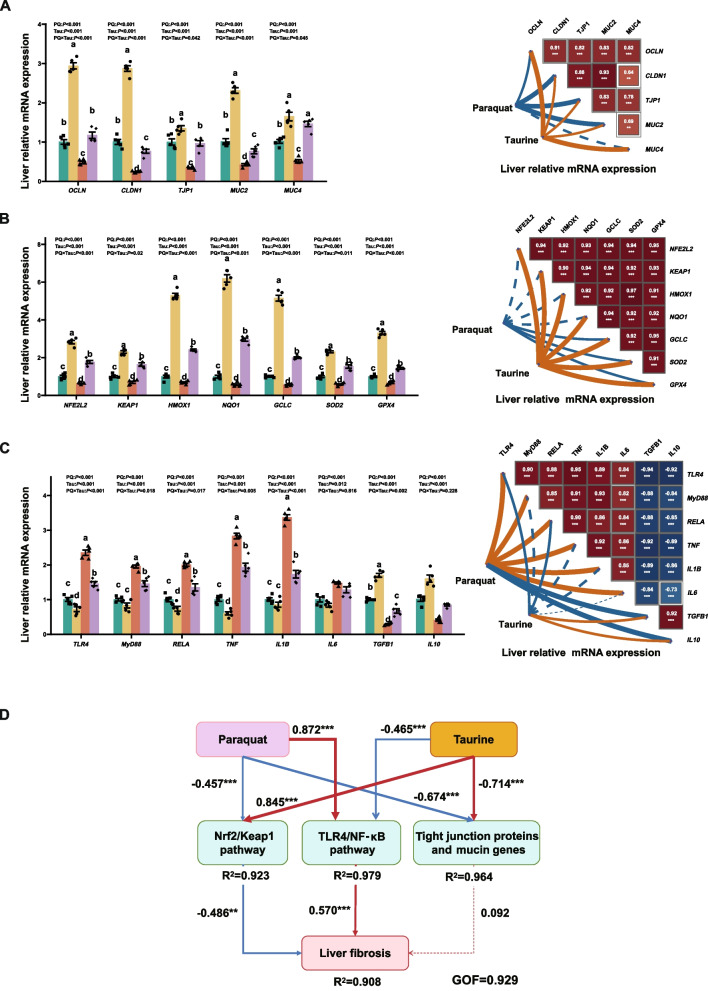
Gene expression in the liver of weaned piglets (*n* = 5). **A** The mRNA expression of tight junction proteins and mucin and their correlations with Tau or PQ. **B** The mRNA expression related to the antioxidant pathway and their correlations with Tau or PQ. **C** The mRNA expression related to the immune pathway and their correlations with Tau or PQ. Means without a common superscript are significantly different (*P* < 0.05). **D** The PLS-PM revealed the regulatory mechanism of dietary Tau and PQ challenge on liver fibrosis in piglets

PQ increased the area of liver fibrosis (*P* < 0.001; Fig. 5A and B). However, Tau reduced this area compared to the diet without Tau supplementation (*P* < 0.001). PQ decreased tight junctions and mucin mRNA expression (*P* < 0.001, Fig. 6A), whereas Tau increased their expression compared to the diet without Tau (*P* < 0.001). A significant PQ × Tau interaction was observed for *OCLN*, *CLDN1*, *TJP1*, *MUC2*, and *MUC4* mRNA (*P* < 0.05), and Tau significantly reversed the PQ-induced downregulation in the expression of these genes.

PQ decreased antioxidant factor levels and increased MDA levels (*P* < 0.001, Fig. 5B). Tau increased antioxidant factor levels and reduced MDA levels compared with the diet without Tau (*P* < 0.05). A significant PQ × Tau interaction was observed for CAT, OH•^−^, and SOD levels (*P* ≤ 0.004), with Tau significantly reversing PQ-induced reductions in hepatic CAT and SOD levels. PQ downregulated the Nrf2 pathway and the mRNA expression of downstream genes (*P* < 0.001, Fig. 6B). Contrastingly, Tau significantly increased their expression compared to the diet without Tau (*P* < 0.001). PQ × Tau significantly interacted with these antioxidant genes in the liver (*P* < 0.02), and Tau reversed the PQ-induced downregulation in the expression of these genes. PQ upregulated the expression of *TLR4*, *MyD88*, *RELA*, *TGFB1*, and *IL10* mRNA (*P* < 0.001, Fig. 6C). Tau suppressed the inflammatory pathway and pro-inflammatory cytokine expression, while enhancing anti-inflammatory cytokine expression compared to the diet without Tau (*P* ≤ 0.012).

PLS-PM was used to analyze the relationship between PQ, Tau, and liver fibrosis (Fig. 6D). PQ and Tau primarily affected liver fibrosis through three key nodes: the Nrf2/Keap1 pathway, the TLR4/NF-κB pathway, and tight junction proteins and mucin genes. PQ negatively affected the Nrf2/Keap1 pathway (β = −0.457) and the expression of tight junction proteins and mucin genes (β = −0.674). Contrastingly, it positively affected the TLR4/NF-κB pathway (β = 0.872); Tau had the opposite effects (*P* < 0.001). The TLR4/NF-κB pathway (β = 0.570) directly promoted liver fibrosis, whereas the Nrf2/Keap1 pathway (β = −0.486) inhibited it (*P* < 0.01).

### Effects of dietary Tau levels and PQ challenge on ileal microbiota in piglets

**Fig. 7:**
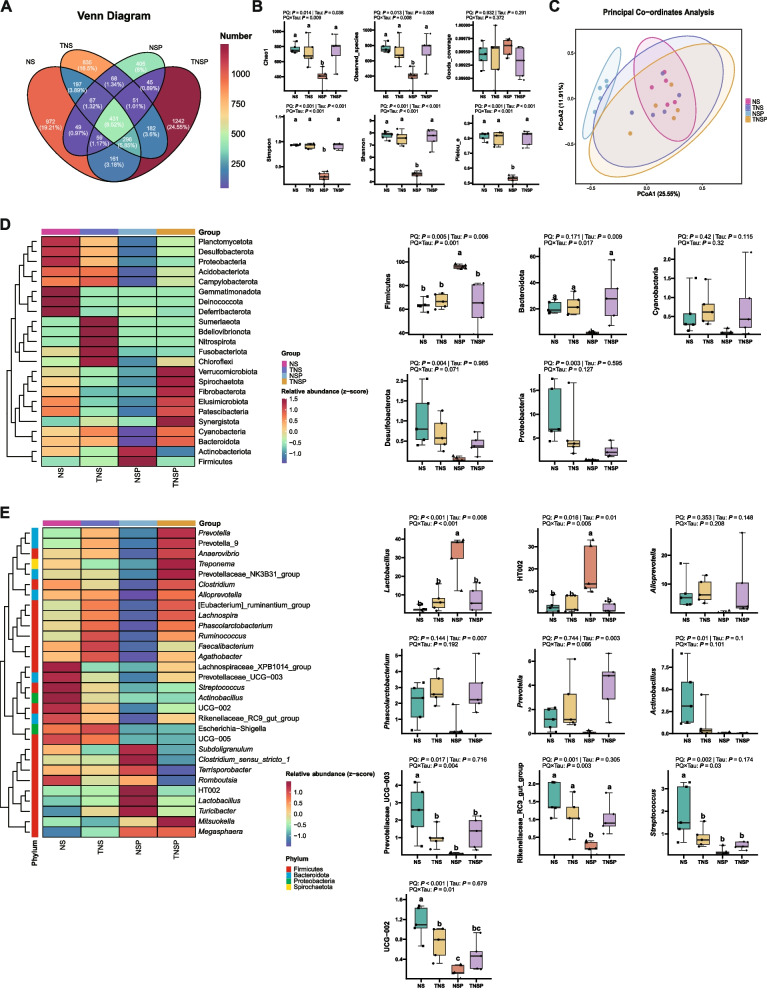
Microbial diversity and composition (*n* = 5). **A** Venn diagram. **B** α-diversity indicators. **C** β-diversity analysis (principal coordinates analysis, PCoA). **D** and **E **The clustering heatmap of community composition for the top 30 species by relative abundance at the phylum level (**D**) and genus level (**E**) was generated. Each row represented a species, and each column represented a group. The heatmap used a gradient from blue to red to reflect changes in abundance from low to high. Species closer to blue indicated lower abundance, while those closer to red indicated higher abundance. Means without a common superscript are significantly different (*P* < 0.05)

In total, 5,060 amplicon sequence variants (ASVs) were identified. Unique and group-specific ASV counts were as follows: TNSP > NS > TNS > NSP (Fig. 7A). PQ reduced all α-diversity indices except goods_coverage (*P* ≤ 0.014, Fig. 7B). In contrast, Tau supplementation increased these α-diversity indices compared to diets without Tau (*P* ≤ 0.05). A significant PQ × Tau interaction was observed for these α-diversity indices (*P* < 0.009); Tau significantly reversed PQ-induced α-diversity decline and stabilized the gut microbiota. Principal coordinate analysis (PCoA) revealed a distinct NSP group, whereas the other groups, especially the Tau-supplemented groups, were similar (Fig. 7C).

We further analyzed changes at the phylum and genus levels. At the phylum level, PQ increased the abundance of Firmicutes and reduced that of Desulfobacterota and Proteobacteria (*P* < 0.005; Fig. 7D). Tau increased the abundance of Bacteroidota compared to diets without Tau supplementation (*P* ≤ 0.009). The PQ × Tau interaction significantly affected the abundances of Firmicutes and Bacteroidota (*P* ≤ 0.004). However, Tau effectively reversed the PQ-induced increase in Firmicutes and decrease in Bacteroidota. At the genus level, PQ reduced the abundance of *Actinobacillus*, Prevotellaceae_UCG-003, Rikenellaceae_RC9_gut_group, *Streptococcus*, and UCG-002. However, it increased the abundances of *Lactobacillus* and HT002 (*P* ≤ 0.017, Fig. 7E). Tau increased the abundance of *Phascolarctobacterium* and *Prevotella* and decreased that of HT002, compared with diets without Tau supplementation (*P* ≤ 0.01). The interaction between PQ and Tau significantly affected *Lactobacillus*, HT002, Prevotellaceae_UCG-003, Rikenellaceae_RC9_gut_group, *Streptococcus*, and UCG-002 (*P* < 0.05). Tau significantly reversed the PQ-induced increase in *Lactobacillus* and HT002 abundance, along with the decrease in Rikenellaceae_RC9_gut_group. The NSP group showed a general decline in species abundance. However, HT002 and *Lactobacillus* were more enriched in the NSP group than in other groups (*P* < 0.05).

**Fig. 8:**
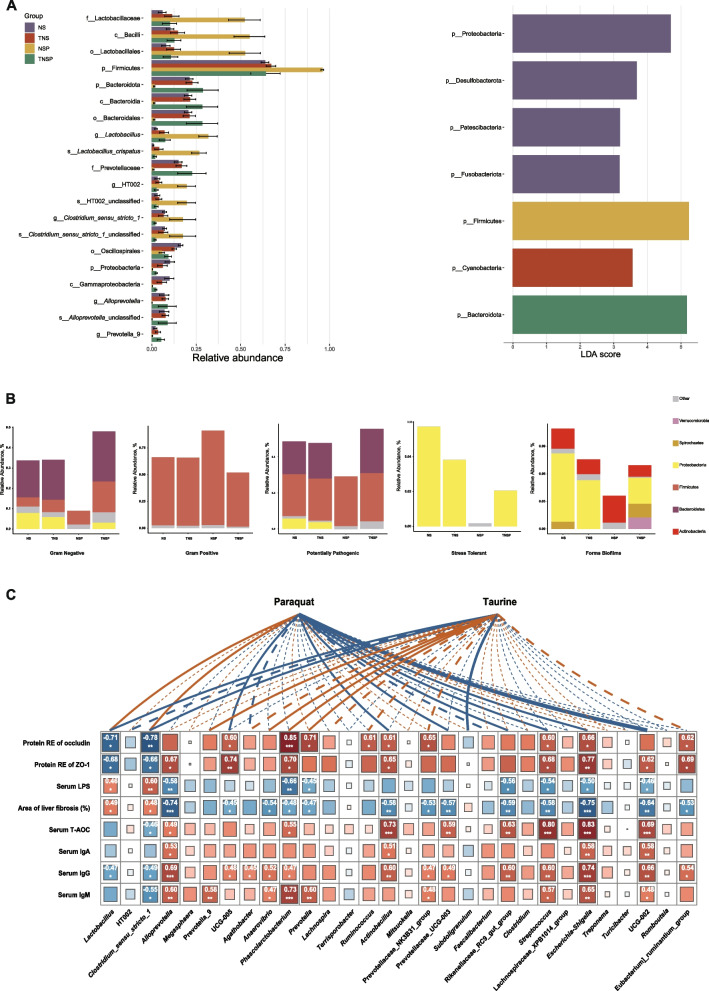
Differential microbial analysis, phenotypic prediction, and microbe–host correlations. **A** The LEfSe differential analysis. The distribution bar chart on the left displayed species with statistically significant differences at various taxonomic levels and their relative abundance. The bar chart on the right used color to represent the group with higher abundance of differentially abundant species at the genus level, while the length of the bars indicated the impact degree of these species on the groups. **B** The bacterial phenotypic analysis was conducted using BugBase, focusing on the enrichment of species associated with five phenotypes. **C** The heatmap displayed the correlations between gut microbial genera and indicators related to intestinal tight junction proteins, liver damage, antioxidant levels, and immune function, as well as the correlations of these genera with Tau or PQ. The colors represented the correlation coefficient *r*, with *, **, and *** indicating *P*-values below 0.05, 0.01, and 0.001, respectively

The LEfSe analysis identified differential species (Fig. 8A), with Firmicutes being the most enriched and showing the highest abundance in the NSP group. At the phylum level, Firmicutes, Bacteroidota, and Proteobacteria were the most influential taxa. Without Tau supplementation, PQ tended to increase the abundance of lactic acid bacteria. However, Tau mitigated the effects of PQ by stabilizing biomarkers. BugBase analysis predicted five bacterial phenotypes: Gram-negative, Gram-positive, pathogenic, stress-tolerant, and biofilm-forming (Fig. 8B). In the absence of Tau supplementation, PQ reduced the abundance of Gram-negative bacteria and increased that of Gram-positive bacteria; Tau reversed this trend. In the absence of PQ, Tau maintains the balance between Gram-negative and Gram-positive bacteria in the gut. PQ reduced the abundance of stress-tolerant and biofilm-forming bacteria, consequently affecting oxidative stress and repair; however, Tau mitigated these effects. The stress-tolerant and biofilm-forming bacteria were mainly Proteobacteria, whereas the potentially pathogenic and Gram-positive bacteria were Firmicutes.

### Regulation of the gut-liver axis by dietary Tau levels and PQ challenge in piglets

Correlation analysis (Fig. 8C) revealed that *Lactobacillus* and *Clostridium_sensu_stricto_1* were positively correlated with PQ and negatively correlated with Tau, whereas genera such as UCG-005, *Actinobacillus*, and *Streptococcus* were negatively correlated with PQ. Some *Prevotella* species were positively correlated with Tau, whereas *Subdoligranulum* was negatively correlated (*P* < 0.05). Furthermore, intestinal Occludin and ZO-1, along with serum T-AOC and IgG, were negatively correlated with *Lactobacillus* and *Clostridium_sensu_stricto_1* but positively correlated with genera such as *Phascolarctobacterium* and *Streptococcus*. Serum LPS levels and liver fibrosis area were positively correlated with *Lactobacillus* and *Clostridium_sensu_stricto_1* and negatively correlated with genera such as *Alloprevotella*, *Phascolarctobacterium*, and *Prevotella* (*P* < 0.05).

**Fig. 9:**
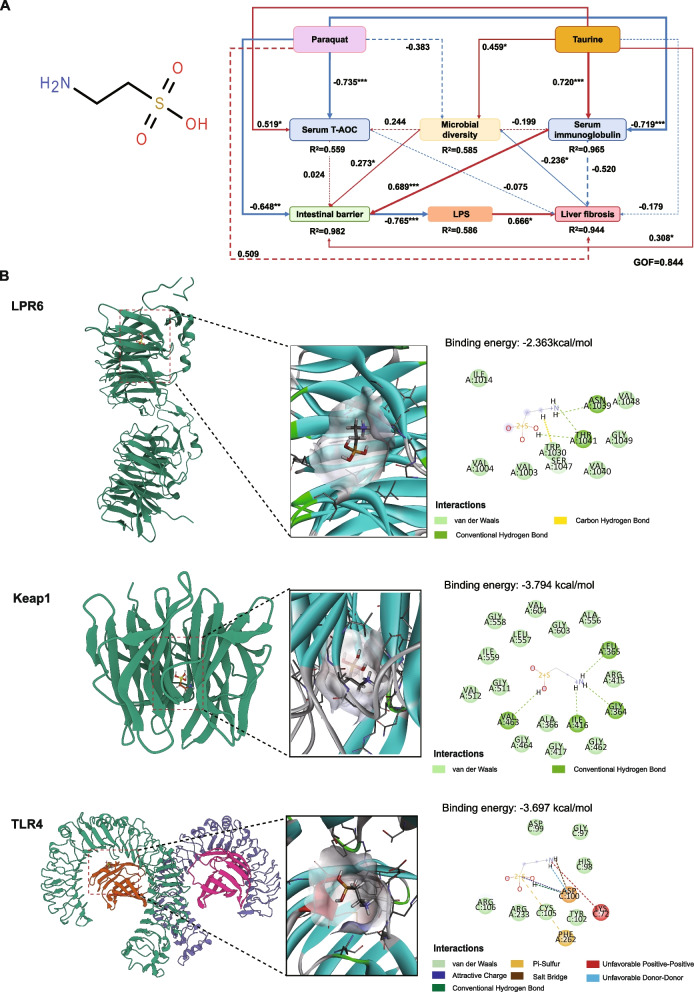
Regulatory mechanisms and molecular interactions related to Tau and PQ. **A** The PLS-PM revealed the regulatory mechanisms of dietary Tau and PQ challenge on the gut-liver axis in piglets. **B** The molecular docking was employed to predict the binding energy and interaction patterns between pathway proteins (LRP6, Keap1, TLR4) and their ligand Tau

PLS-PM analysis revealed the mechanisms through which PQ and Tau regulate key gut-liver axis indicators in piglets (Fig. 9A). PQ reduced serum T-AOC (β = −0.735) and immunoglobulin (β = −0.719) levels, impaired gut barrier function (β = −0.648), and consequently elevated LPS levels (β = −0.765). This exacerbated inflammation and oxidative stress, ultimately inducing liver fibrosis (β = 0.666). Liver damage further altered the gut microbial composition (β = −0.236), disrupted gut homeostasis, and contributed to a vicious gut-liver axis cycle (*P* < 0.05). Moreover, the direct effect of PQ on gut microbiota composition was weak (β = −0.383, *P* > 0.05). In contrast, Tau supplementation markedly improved gut barrier function (β = 0.308), elevated serum T-AOC (β = 0.519) and immunoglobulin (β = 0.720) levels, reduced LPS release, and ultimately decreased liver fibrosis (*P* < 0.05). Additionally, Tau exerted a direct positive effect on gut microbial diversity (β = 0.107, *P* < 0.05).

Molecular docking was used to predict and analyze the binding modes of Tau with key signaling proteins, including LRP6 (involved in mucosal injury repair), Keap1 (an antioxidant pathway protein), and TLR4 (an immune-related protein) (Fig. 9B). The calculated binding energy of Tau with LPR6 was −2.363 kcal/mol. Although this value was moderate compared with that of high-affinity ligands, it highlights the potential role of Tau as a modulator of LPR6 function. Tau interacted with several key residues within the LPR6 binding pocket through hydrogen bonding and hydrophobic interactions. The three-dimensional structure revealed that Tau was located in a well-defined hydrophilic binding pocket surrounded by amino acid residues. This created a favorable electrostatic environment, further supporting its binding. In addition, residues such as Asn1039 and Gly1049 formed a supportive network with weak interactions, consequently stabilizing the overall conformation of the complex. The docking analysis of Tau with Keap1 yielded a binding energy of −3.794 kcal/mol, indicating moderate affinity. Hydrogen bonds formed with key residues such as ILE416 and ARG415 likely played crucial roles in the orientation of Tau and stabilization of the interaction. Van der Waals forces further enhanced stability through complementary hydrophobic contacts within the binding site. Molecular docking of Tau with TLR4 yielded a binding energy of −3.697 kcal/mol. Key interactions between Tau and residues within the TLR4 binding pocket included hydrogen bonds, van der Waals forces, and electrostatic interactions. Despite favorable binding contributions, minor steric clashes and unfavorable donor-donor or positive-positive interactions with LYS72 and PHE262 slightly reduced binding efficiency. Their binding suggests that Tau acts as a weak binder, with its physiological relevance possibly depending on secondary mechanisms rather than direct binding.

## Discussion

In this study, we aimed to elucidate the protective effects of Tau against PQ-induced oxidative stress and gut-liver axis injury in weaned piglets. Tau is a sulfur-containing β-amino acid that interacts with macromolecules (proteins, lipids, and nucleic acids) to regulate various biological functions [23]. It modulates oxidative stress [24], maintains cellular homeostasis [25], and promotes tissue repair [26]. Although previous studies have demonstrated the antioxidant and anti-inflammatory properties of Tau in rodents and poultry [27–29], its specific contribution to gut-liver axis health in weaned piglets remains largely unexplored. Our study addresses this knowledge gap; Tau alleviated PQ-induced gut-liver axis damage (impaired intestinal barrier function, liver injury, and altered gut microbial composition) by modulating the Nrf2/Keap1 and TLR4/NF-κB signaling pathways. This amino acid also improved systemic oxidative and immune functions, leading to enhanced ADG and FCR activity. Moreover, our study highlights the interaction between Tau and key molecular signaling pathways, further elucidating the mechanisms underlying biomolecular interactions. We further clarified the relationships between PQ exposure, Tau intervention, gut-liver axis function, and systemic oxidative stress using PLS-PM.

### Tau improved intestinal barrier function in piglets

The intestinal barrier is a critical component of the gut-liver axis. It maintains systemic homeostasis and defense against external pathogens [30]. In this study, PQ exposure resulted in decreased expression of tight junction proteins, villus disruption, crypt elongation, and increased intestinal permeability, indicating impaired barrier function. Tau supplementation significantly mitigated these adverse effects. Consistent with previous findings [31], Tau was positively correlated with intestinal VH and goblet cell numbers, highlighting its role in supporting epithelial integrity and mucosal protection.

The ability of Tau to improve the intestinal barrier function was attributed to its antioxidant and anti-inflammatory properties. In the present study, PQ exposure elevated oxidative stress markers and reduced antioxidant enzyme activity. In contrast, Tau substantially restored the activities of these enzymes and upregulated the expression of genes related to the Nrf2/Keap1 pathway. The protective effect of Tau against oxidative stress primarily stems from its ability to reduce ROS production and activate endogenous antioxidant systems. Moreover, the presence of Tau significantly decreases the likelihood of protein mistranslation in the mitochondrial electron transport chain [32]. In contrast, this amino acid induces the expression of antioxidant enzyme genes by activating the Keap1-Nrf2/ARE signaling pathway [33]. Additionally, we theorized that Tau is a key pH regulator within the mitochondria, contributing to redox homeostasis by acting on the GSH/GSSG system in the mitochondrial redox network [34].

PQ exposure elevated the levels of inflammatory markers in the intestinal mucosa, whereas Tau alleviated inflammation by inhibiting the expression of genes related to TLR4/NF-κB pathway. These properties have been exploited to develop hydrogel patches that reduce oxidative damage and inflammation by inhibiting ROS/NF-κB signaling in macrophages, thereby creating a favorable immune microenvironment for tissue repair [35]. Notably, Qi et al. [14] used PQ-induced in vitro and in vivo oxidative stress models to identify *MyD88* as a novel target of intestinal oxidative stress. Our study also demonstrated that *MyD88*, a key adaptor in the TLR4 signaling pathway, mediates downstream inflammatory responses and oxidative stress, consequently linking the activation of innate immunity to oxidative damage.

Tau maintains epithelial renewal and tight junction integrity. In our study, it upregulated the expression of the intestinal stem cell marker *LGR5*, enhanced the expression of genes related to intestinal cell renewal and proliferation, and significantly increased the expression of the scaffold protein ZO-1. This was closely linked to the activation of the Wnt/β-catenin signaling pathway [36]. Based on the findings of Kuo et al. [37], which demonstrated that ZO-1 made a key contribution to Wnt signaling and mitotic spindle orientation through tight, junction-independent mechanisms, we theorized that Tau supplementation further promotes the upregulation of epithelial proliferation and the successful completion of mitosis. Additionally, Tau upregulated the expression of tight junction proteins, enhancing intestinal barrier integrity.

### Tau alleviated liver injury and improved liver function in piglets

As the second line of defense after the intestinal barrier [38], the liver is another primary target organ for PQ toxicity. In this study, PQ-induced oxidative stress and inflammation led to liver damage in piglets, including compromised barrier integrity, marked expansion of fibrosis, and dysregulated antioxidant and immune functions. Dietary Tau alleviated these effects by enhancing integrity of the liver barrier, reducing fibrosis, and improving antioxidant and immune capacities, consistent with the findings of Ji et al. [39].

Consistent with previous studies [40], Tau significantly enhanced the liver antioxidant defense by activating the Nrf2/Keap1 pathway and increasing the activity of antioxidant enzymes, thereby counteracting PQ-induced oxidative stress. Cysteine is the synthetic precursor for both Tau and GSH. Exogenous Tau reduces the demand for endogenous synthesis, thereby conserving cysteine for GSH production [41]. This was supported by a significant elevation in liver GSH-Px activity in the Tau groups, suggesting that Tau enhances antioxidant capacity by activating the GSH–GSH-Px system. In addition, Tau regulates inflammation by suppressing the secretion of pro-inflammatory cytokines and promoting that of anti-inflammatory cytokines. This amino acid can also regulate immune signaling pathways, including NF-κB and MAPK, which are often overactivated during liver damage [42]. Recent studies have revealed that tumor cells promote progression by overexpressing the Tau transporter SLC6A6 to compete with CD8⁺ T cells for Tau, leading to T cell exhaustion and dysfunction; supplementation with Tau reverses this process [43]. Therefore, we hypothesized that Tau alleviates inflammatory liver injury by downregulating pro-inflammatory pathways, such as TLR4/NF-κB, and supporting the appropriate activation of CD8⁺ T cells. These findings further support the potential use of Tau as a hepatoprotectant. Moreover, Tau upregulates the expression of tight junction proteins to strengthen the liver barrier and prevent the translocation of harmful substances and bacteria via the bile or blood [44], thereby contributing to reduced fibrosis and overall liver injury.

### Regulation of intestinal microbiota by Tau in piglets

In this study, dietary Tau significantly modulated ileal microbial diversity and composition in piglets, particularly under PQ-induced oxidative stress. Tau significantly increased microbial α-diversity and restored the PQ-induced reduction in species richness and evenness in the TNSP group, suggesting its ability to alleviate acute oxidative stress by regulating microbial diversity. Moreover, PCoA revealed a similar microbial composition among the other three groups, excluding the NSP group, further supporting the role of Tau in microbial homeostasis.

Phylum-level analysis showed that PQ challenge markedly reduced the abundance of Bacteroidota and Proteobacteria, whereas Tau restored the levels of Bacteroidota and maintained Firmicute abundance. These findings suggest that the gut microbiota responds to Tau-mediated regulation of the gut-liver axis. Tau-mediated restoration of Bacteroidota and Proteobacterial abundance may enhance intestinal metabolic activity, subsequently improving liver antioxidant and immune functions. We hypothesized that the gut microbiota responds to the regulation of the gut-liver axis by Tau. Similarly, specific microbial taxa affect gut-liver axis function by modulating bile acid metabolism and antioxidant pathways [45].

At the genus level, probiotics such as *Lactobacillus* and HT002 were significantly enriched in the NSP group; however, their abundance was restored after Tau supplementation in the TNSP group. This selective modulation of probiotics may have alleviated inflammation by activating the Toll-like receptor signaling pathway (TLR4/NF-κB) [46]. Akhtar et al. [47] reported the protective effects of probiotics against host inflammation and oxidative stress. For example, *Lactobacillus* alleviates oxidative damage by secreting antioxidant enzymes (e.g., SOD and CAT) and producing metabolites such as lactic acid and short-chain fatty acids [48]. This genus also generates antioxidant-related metabolites, including lactic acid and exopolysaccharides [49]. This might provide an ecological niche advantage under stressful conditions. Oxidative stress was markedly reduced and the intestinal environment returned to near homeostasis after Tau supplementation. This restored the abundance of *Lactobacillus* and HT002 to near-normal levels. These findings indicate that Tau mitigates microbial stress responses by restoring intestinal stability.

BugBase analysis showed that Tau maintained a balance between Gram-negative and Gram-positive bacteria under normal conditions. Oxidative stress favors the proliferation of Gram-positive bacteria, whereas Tau supplementation effectively reverses this imbalance. Tau likely restore microbial equilibrium by adjusting the intestinal redox status or by activating ROS-scavenging mechanisms [50]. In addition, the reduction in microbial groups associated with stress tolerance and biofilm formation indicated that Tau may repair intestinal injury by suppressing opportunistic pathogens associated with oxidative stress and biofilm development, such as Proteobacteria [51].

### Mechanisms by which Tau regulated gut-liver axis function in piglets

In this study, dietary Tau ameliorated PQ-induced gut-liver axis damage and systemic oxidative stress. Molecular docking revealed that Tau exhibited binding affinity to LRP6, Keap1, and TLR4 proteins. This suggests the potential mechanisms by which Tau activated the Wnt/β-catenin, Nrf2/Keap1, and TLR4/NF-κB signaling pathways. PLS-PM results indicated that Tau positively affected the Nrf2/Keap1 pathway and negatively modulated the TLR4/NF-κB pathway, thereby improving intestinal barrier function and alleviating liver fibrosis. Furthermore, PLS-PM showed that Tau supplementation exerts a positive effect on serum T-AOC (β = 0.519, *P* < 0.05) and immunoglobulin levels (β = 0.720, *P* < 0.001), highlighting its key role in the alleviation of oxidative stress and its potential for immunomodulation.

The regulatory effect of Tau on the gut-liver axis is characterized by a bidirectional crosstalk mechanism, with its core effects manifested in the protection of the intestinal barrier and the modulation of liver immune and metabolic functions. The intestinal mucosal barrier serves as the first line of defense against exogenous substances under normal physiological conditions. The liver, acting as the second line of defense, not only eliminates antigens and cytokines that escape mucosal immune surveillance but also modulates cellular networks to propagate immune tolerance to other organs [52]. However, harmful substances such as LPS enter the liver via the portal vein, activate intrahepatic immune cells, and trigger the LPS-CD14-TLR4 signaling pathway when the intestinal barrier is compromised, thereby releasing pro-inflammatory mediators that induce inflammation and injury in both the liver and intestine. Liver injury further impairs intestinal function by reducing Kupffer cell phagocytosis, disrupting hemodynamics, and hindering immune protein synthesis, thereby forming a vicious cycle [53]. In this study, Tau disrupted this vicious cycle through multiple mechanisms, thereby exerting a bidirectional protective effect on the gut-liver axis. From an intestinal perspective, Tau enhances barrier function, thereby reducing LPS leakage and its inflammatory burden on the liver. We hypothesized that Tau binds to LRP6 to enhance the Wnt signaling pathway activity, improve the expression of tight junction proteins, and reinforce intestinal barrier integrity. From a hepatic perspective, this amino acid activates antioxidant and immune-regulatory pathways, indirectly protects liver sinusoidal endothelial cells, and ultimately contributes to the strengthening of hepatic tight junctions and barrier function [54]. Molecular docking suggested that Tau might binds to Keap1 in both the intestinal mucosa and liver, thereby inhibiting Nrf2 degradation, promoting its nuclear translocation, and activating the expression of antioxidant genes and proteins. This consequently alleviates oxidative stress. We further theorized that Tau binding to TLR4 inhibits the NF-κB signaling pathway and reduces the release of pro-inflammatory cytokines. In addition, Tau may modulate bile acid metabolism [55], consequently optimizing the composition and function of the gut microbiota and further promoting synergistic protection between the gut and liver. This complex signaling pathway regulation occurred through the gut-liver axis, forming a systemic protective mechanism that improved growth performance in piglets to a certain extent. However, future studies should investigate the long-term effects of Tau and its potential synergistic interactions with other nutritional interventions, such as probiotics and prebiotics, to further optimize the gut-liver axis function and improve animal productivity. Furthermore, exploring the precise binding mechanisms between Tau and TLR4, Keap1, and LRP6 could clarify the multi-targeted actions of this amino acid.

## Conclusion

**Fig. 10:**
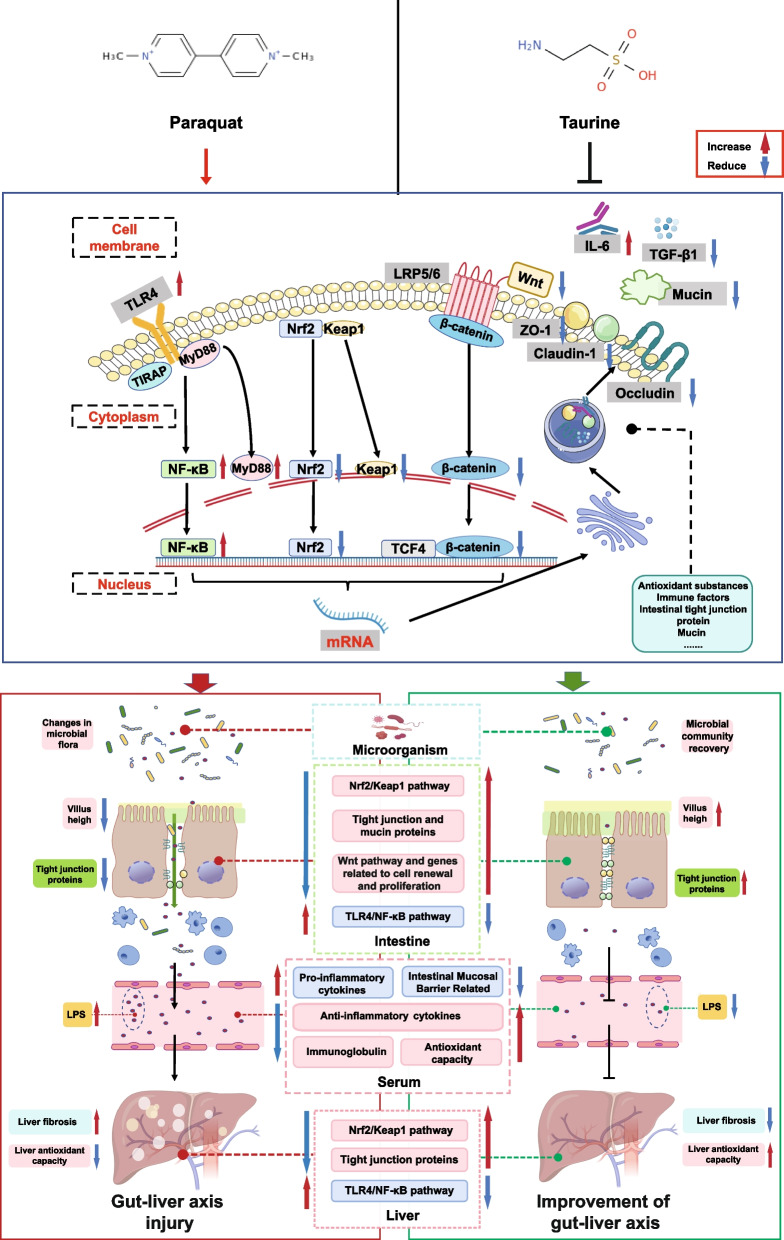
The mechanism through which Tau alleviated PQ-induced oxidative stress and gut-liver damage in weaned piglets by regulating the Nrf2/Keap1 and TLR4/NF-κB signaling pathways

To our knowledge, this study is the first to systematically demonstrate that dietary supplementation with 0.4% (w/w) Tau prevents PQ-induced oxidative stress and gut-liver axis damage by regulating the Nrf2/Keap1 and TLR4/NF-κB signaling pathways in weaned piglets (Fig. 10). Tau enhanced antioxidant and immune defenses of the intestine and liver by activating Nrf2/Keap1 pathway and inhibiting TLR4/NF-κB pathway, thereby strengthening intestinal barrier function (including the gut microbiota), reducing LPS translocation, and alleviating liver injury. These improvements provided positive feedback to the gut-liver axis circulation, ultimately supporting better growth performance in piglets. Thus, these findings highlight its potential use in nutritional intervention.

## Supplementary information

Additional file 1: Table S1a Composition and nutritional levels of the basal diet for weaned piglets. Table S1b Primer sequences for gene amplification.

## Data Availability

The raw data for 16S rDNA sequencing can be accessed at NCBI with the accession number PRJNA1215809 (https://www.ncbi.nlm.nih.gov/bioproject/1215809). Additional raw data supporting the findings of this study are available from the authors upon request.

## Abbreviations

BW: Body weight
ADG: Average daily gain
ADFI: Average daily feed intake
ASVs: Amplicon sequence variants
CAT: Catalase
CD: Crypt depth
CLDN1: Claudin-1
FCR: Feed conversion ratio
GSH-Px: Glutathione peroxidase
IL-1β: Interleukin 1β
IL-6: Interleukin 6
IL-10: Interleukin 10
Keap1: Kelch-like ECH-associated protein 1
LGR5: Leucine-rich repeat-containing G-protein coupled receptor 5
LPS: Lipopolysaccharide/Endotoxin
LRP6: Low-density lipoprotein receptor-related protein 6
MDA: Malondialdehyde
MUC2: Mucin 2
MUC4: Mucin 4
MyD88: Myeloid differentiation primary response 88
NF-κB: Nuclear factor kappa-light-chain-enhancer of activated B cells
NO: Nitric oxide
NSP: Piglets were fed a basal diet without Tau and injected with PQ
NS: Piglets were fed a basal diet without Tau and injected with saline
Nrf2: Nuclear factor erythroid 2-related factor 2
O_2_•^−^: Superoxide anion scavenging activity
OCLN: Occludin
OH•^−^: Hydroxyl radical scavenging activity
PCoA: Principal coordinate analysis
PDB: Protein data bank
PGE2: Prostaglandin E2
PLS-PM: Partial least squares path modeling
PQ: Paraquat
ROS: Reactive oxygen species
SOD: Superoxide dismutase
T-AOC: Total antioxidant capacity
Tau: Taurine
TCF4: Transcription factor 4
TGF-β1: Transforming growth factor β1
TJP1: Tight junction protein 1
TLR4: Toll-like receptor 4
TNF-α: Tumor necrosis factor α
TNS: Piglets were fed a basal diet with 0.4% Tau added and injected with saline
TNSP: Piglets were fed a basal diet with 0.4% Tau added and injected with PQ
VH: Villus height
ZO-1: Zonula occludens-1
