# Supplementary material for: Taurine alleviated paraquat-induced oxidative stress and gut-liver axis damage in weaned piglets by regulating the Nrf2/Keap1 and TLR4/NF-κB signaling pathways
Source: J Anim Sci Biotechnol. 2025 Aug 18;16:117. doi: 10.1186/s40104-025-01244-3 (PMC12359926; doi:10.1186/s40104-025-01244-3)
Supplement: Supplementary file 1 — Additional file 1: Table S1a Composition and nutritional levels of the basal diet for weaned piglets. Table S1b Primer sequences for gene amplification. [file 40104_2025_1244_MOESM1_ESM.docx]

**Table S1a** Composition and nutritional levels of the basal diet for weaned piglets (DM basis, %)

| **Ingredients** | **Content, %** | **Nutritional level** | **Content** |
| --- | --- | --- | --- |
| Corn (CP: 7.6%) | 43.29% | Crude fat^c^ | 5.41% |
| Cracked rice (CP: 7%) | 20.20% | Crude fiber^c^ | 2.11% |
| Bran | 2.02% | Crude ash^c^ | 3.73% |
| 46% soybean meal | 6.06% | Crude protein^c^ | 17.02% |
| Extruded soybeans | 5.05% | Digestible protein^b^ | 14.38% |
| Soy protein concentrate | 5.05% | Digestible energy^b^ | 3,482 kcal/kg |
| Fermented soybean meal | 4.04% | Net energy^b^ | 2,482 kcal/kg |
| Egg yolk powder | 2.53% | Calcium^c^ | 0.49% |
| Low-protein whey powder | 2.53% | Total phosphorus^c^ | 0.52% |
| Coconut oil powder (50% fat) | 2.02% | Available phosphorus^b^ | 0.31% |
| Glucose | 1.52% | SID lysine^d^ | 1.22% |
| Sucrose | 1.52% | SID methionine^d^ | 0.49% |
| Limestone powder | 0.51% | SID cysteine^d^ | 0.23% |
| Dicalcium phosphate | 1.01% | SID methionine + cysteine^d^ | 0.72% |
| Sodium chloride | 0.25% | SID threonine^d^ | 0.84% |
| 0.2% trace elements for piglets | 0.20% | SID tryptophan^d^ | 0.26% |
| Complex vitamins for piglets | 0.04% | SID valine^d^ | 0.81% |
| Benzoic acid | 0.40% | SID arginine^d^ | 0.97% |
| 98% L-lysine | 0.61% | SID histidine^d^ | 0.40% |
| DL-methionine (99%) | 0.25% | SID isoleucine^d^ | 0.62% |
| L-threonine (98.5%) | 0.30% | SID leucine^d^ | 1.20% |
| L-tryptophan (98%) | 0.10% | SID phenylalanine^d^ | 0.68% |
| L-Valine (99%) | 0.15% | SID tyrosine^d^ | 0.47% |
| L-Isoleucine (99%) | 0.05% | SID (phenylalanine + tyrosine)^d^ | 1.15% |
| Mildew inhibitor | 0.10% |  |  |
| Sweetener | 0.02% |  |  |
| 50% Choline chloride | 0.10% |  |  |
| Complex enzymes for piglets | 0.06% |  |  |

^a^ Each kg of the premix diet provided the following components: V_A_: 11,400 IU; VD_3_: 3,200 IU; VE: 48 mg; VK_3_: 3.2 mg; VB_1_: 4.5 mg; VB_2_: 11.2 mg; VB_6_: 4.8 mg; VB_12_: 56 mg; D-biotin: 0.24 mg; folic acid: 2.4 mg; niacin: 56 mg; D-pantothenic acid: 24 mg; Cu: 90.48 mg; Fe: 132.36 mg; Zn: 68.9 mg; Mn: 31.8 mg; Co: 0.72 mg; I: 0.768 mg; Se: 0.344 mg

^b^ Calculated values

^c^ Analysed values

^d^ Values for the concentrations of SID AA in diets were calculated using standardised ileal digestible coefficients for the various ingredients provided by NRC (2012)

**Table S1b** Primer sequences for gene amplification

| **Gene name^a^** | **NCBI ID** | **Sequence of primer (5’→3’)** | **Product size, bp** | **Tm^b^, ℃** |
| --- | --- | --- | --- | --- |
| *GAPDH* | [NM_001206359.1](https://www.ncbi.nlm.nih.gov/entrez/viewer.fcgi?db=nucleotide&id=329744641) | F: CGAGATCCCGCCAACATCAA | 109 | 58.0 |
|  |  | R: CCCCACCCTTCAAGTGAGC |  | 59.3 |
| *OCLN* | [NM_001163647.2](https://www.ncbi.nlm.nih.gov/entrez/viewer.fcgi?db=nucleotide&id=402746997) | F: CAGTGGTAACTTGGAGGCGT | 104 | 57.0 |
|  |  | R: CCGTCGTGTAGTCTGTCTC |  | 55.0 |
| *CLDN1* | NM_001244539.1 | F: GGACAAAACCGTGTGGGAAC | 200 | 57.1 |
|  |  | R: CACTTTCCCGTTGGACGAGT |  | 55.3 |
| *TJP1* | [XM_021098827.1](https://www.ncbi.nlm.nih.gov/entrez/viewer.fcgi?db=nucleotide&id=1191808307) | F: CGTGTCAACGCCACTATCA | 90 | 55.1 |
|  |  | R: TTGTCTTCCAAAGCCCCT |  | 53.7 |
| *MUC2* | XM_021082584.1 | F: CTGTGCGACTACAACTTCGC | 139 | 56.7 |
|  |  | R: AGATGGTGTCGTCCTTGACC |  | 57.1 |
| *MUC4* | [XM_021068273.1](https://www.ncbi.nlm.nih.gov/entrez/viewer.fcgi?db=nucleotide&id=1191824461) | F: AGGATGCCCAATGGCTCTACT | 96 | 60.98 |
|  |  | R: AAGGAGGCTGGTTCCGTTGAT |  | 61.73 |
| *VIL1* | XM_001925167.6 | F: ACGGCGCAGGAGTATCTCAA | 187 | 58.5 |
|  |  | R: ACGGCGCAGGAGTATCTCAA |  | 59.1 |
| *LGR5* | [NM_001315762.1](https://www.ncbi.nlm.nih.gov/entrez/viewer.fcgi?db=nucleotide&id=937575156) | F: GCCTCGGAATCCTCCGTTTT | 105 | 58.0 |
|  |  | R: CAGGCTGGAGGAGGGAGTTT |  | 59.8 |
| *BMI1* | [NM_001285971.1](https://www.ncbi.nlm.nih.gov/entrez/viewer.fcgi?db=nucleotide&id=552953714) | F: CCAGAACAGATTGGATCGGA | 90 | 54.2 |
|  |  | R: GCTGCTGGGCACCGTAA |  | 59.3 |
| *PCNA* | NM_001291925.1 | F: CCTGTGCAAAAGATGGAGTG | 187 | 54.0 |
|  |  | R: GGAGAGAGTGGAGTGGCTTT |  | 56.9 |
| *CTNNB1* | [NM_214367.1](https://www.ncbi.nlm.nih.gov/entrez/viewer.fcgi?db=nucleotide&id=47523791) | F: GGTTAAACTCCTACATCCACCA | 148 | 53.9 |
|  |  | R: AGCACGAACCAGCAACTGA |  | 57.3 |
| *TCF4* | [XM_021093431.1](https://www.ncbi.nlm.nih.gov/entrez/viewer.fcgi?db=nucleotide&id=1191806826) | F: CCGGTCTTACTTTCCGCCAA | 285 | 57.9 |
|  |  | R: CTCGTCGTCGGACTTGATCTC |  | 57.7 |
| *CCND1* | XM_021082686.1 | F: CTCAAGTGGAACCTGGCGG | 235 | 59.4 |
|  |  | R: AGGACAGGAAGCTGTTGGAACT |  | 58.5 |
| *NFE2L2* | [XM_005671981.3](https://www.ncbi.nlm.nih.gov/entrez/viewer.fcgi?db=nucleotide&id=1191841844) | F: TGCAGCTTTTGGCAGAGACA | 119 | 57.5 |
|  |  | R: AGGAGCAATGAAGACTGGGC |  | 57.8 |
| *KEAP1* | NM_001114671 | F: AAACCGCCTCAACTCAGCAG | 148 | 58.4 |
|  |  | R: CTGGTCCTGACCATCGTAGC |  | 58.1 |
| *HMOX1* | NM_001004027.1 | F: TACCGCTCCCGAATGAACAC | 209 | 57.6 |
|  |  | R: GTCACGGGAGTGGAGTCTTG |  | 58.2 |
| *NQO1* | NM_001159613.1 | F: TATCCTCCTCTGGCCAATTC | 81 | 53.8 |
|  |  | R: AGGCGTTTCTTCCACTCTTC |  | 54.9 |
| *GCLC* | XM_021098556.1 | F: GATCCTCCAGTTCCTGCACA | 87 | 57.2 |
|  |  | R: GAGAGAGAACCAACCTCGTCG |  | 57.7 |
| *SOD2* | NM_214127.2 | F: GGACAAATCTGAGCCCTAACG | 159 | 55.8 |
|  |  | R: CCTTGTTGAAACCGAGCC |  | 54.3 |
| *GPX4* | NM_214407.1 | F: TCACCAATGTGGCCTCTCAA | 109 | 56.5 |
|  |  | R: CAAGGGAAGGCCAGAATCCG |  | 59.1 |
| *TLR4* | NM_001293316.1 | F: TCATCCAGGAAGGTTTCCAC | 234 | 54.5 |
|  |  | R: TGTCCTCCCACTCCAGGTAG |  | 58.7 |
| *MyD88* | NM_001099923.1 | F: CAGCATCCCTTGGATGTCAGG | 101 | 58.6 |
|  |  | R: GGATATCGCTGGGGCAGTAG |  | 58.1 |
| *RELA* | NM_001114281.1 | F: CGGGGACTACGACCTGAATG | 117 | 58.1 |
|  |  | R: GCACGGTTGTCAAAGATGGG |  | 57.2 |
| *TNF* | NM_214022.1 | F: TGGCCCAAGGACTCAGATCA | 113 | 58.6 |
|  |  | R: GGCATTGGCATACCCACTCT |  | 57.9 |
| *IL1B* | [NM_214055.1](https://www.ncbi.nlm.nih.gov/entrez/viewer.fcgi?db=nucleotide&id=47522925) | F: GCAGTGGAGAAGCCGATGAA | 148 | 57.9 |
|  |  | R: TAGACTGCACGTTGGCATCA |  | 56.6 |
| *IL6* | NM_214399.1 | F: TGGCTACTGCCTTCCCTACC | 153 | 59.6 |
|  |  | R: CACACATCTCCTTTCTCATTGC |  | 53.8 |
| *TGFB1* | NM_214015.2 | F: GGCCGTACTGGCTCTTTACA | 126 | 57.4 |
|  |  | R: CCGCTTTCCAGCATTAGCAC |  | 57.3 |
| *IL10* | NM_214041.1 | F: CTGCATCCACTTCCCAACCA | 77 | 58.0 |
|  |  | R: AGAAACTCTTCACTGGGCCG |  | 57.6 |

^a^*GAPDH*, glyceraldehyde-3-phosphate dehydrogenase; *OCLN*, occludin; *CLDN1*, claudin-1; *TJP1*, tight junction protein 1; *MUC2*, mucin 2; *MUC4*, mucin 4; *VIL1*, villin 1; *LGR5*, leucine-rich repeat-containing G-protein coupled receptor 5; *BMI1*, B lymphoma Mo-MLV insertion region 1 homolog; *PCNA*, proliferating cell nuclear antigen; *CTNNB1*, catenin beta 1; *TCF4*, transcription factor 4; *CCND1*, cyclin D1; *NFE2L2*, nuclear factor, erythroid 2 like 2; *KEAP1*, kelch-like ECH-associated protein 1; *HMOX1*, heme oxygenase 1; *NQO1*, NAD(P)H quinone dehydrogenase 1; *GCLC*, glutamate-cysteine ligase catalytic subunit; *SOD2*, superoxide dismutase 2, mitochondrial; *GPX4*, glutathione peroxidase 4; *TLR4*, toll-like receptor 4; *MyD88*, myeloid differentiation primary response 88; *RELA*, RELA proto-oncogene, NF-κB subunit; *TNF*, tumor necrosis factor; *IL1B*, interleukin 1 beta; *IL6*, interleukin 6; *TGFB1*, transforming growth factor beta 1; *IL10*, interleukin 10.

^b^Tm, melting temperature.
